# Understanding the Vicious Circle of Pain, Physical Activity, and Mental Health in Lipedema Patients—A Response Surface Analysis

**DOI:** 10.3390/jcm12165319

**Published:** 2023-08-16

**Authors:** Marie-Luise Aitzetmüller-Klietz, Lena Busch, Matthias Hamatschek, Matthias Paul, Carsten Schriek, Philipp Wiebringhaus, Matthias Aitzetmüller-Klietz, Maximilian Kückelhaus, Tobias Hirsch

**Affiliations:** 1 Department of Plastic, Reconstructive and Aesthetic Surgery, Hand Surgery, Fachklinik Hornheide, 48157 Muenster, Germany; 2 Plastic and Reconstructive Surgery, Institute of Musculoskeletal Medicine, Westfalian Wilhelms-University, 48149 Muenster, Germany; 3 Division of Plastic and Reconstructive Surgery, Department of Trauma, Hand and Reconstructive Surgery, University Hospital Muenster, 48149 Muenster, Germany; 4 Academy for Diagnostics and Prevention, 48149 Muenster, Germany; 5 Department of Cardiovascular Medicine, Division Steinfurt, University Hospital Muenster, 48565 Steinfurt, Germany

**Keywords:** lipedema, pain, mental health, physical activity, depression

## Abstract

Lipedema is a widespread disease with painful accumulations of subcutaneous fat in the legs and arms. Often, obesity co-occurs. Many patients suffer from impairment in mobility and mental health. Obesity and mental health in turn can be positively influenced by physical activity. In this study, we aimed to examine the interrelations between pain and physical activity on mental health in lipedema patients. In total, 511 female lipedema patients (age M = 40.16 ± 12.45 years, BMI M = 33.86 ± 7.80 kg/m^2^) filled in questionnaires measuring pain, physical activity, and mental health (PHQ-9; WHOQOL-BREF with subscales mental, physical, social, environmental, and overall health). Response surface analyses were calculated via R statistics. The explained variance was high for the model predicting depression severity (R^2^ = 0.18, *p* < 0.001) and physical health (R^2^ = 0.30, *p* < 0.001). Additive incongruence effects of pain and physical activity on depression severity, mental, physical, and overall health were found (all *p* < 0.001). In our study, physical activity and pain synergistically influenced physical, mental, and overall health. The pain not only led to low mental health but also interfered with the valuable potential of engaging in physical activity in lipedema patients.

## 1. Introduction

Lipedema is a widespread disease characterized by large accumulations of subcutaneous fat that primarily affects the legs and limbs. The distribution pattern is mostly bilaterally and symmetrically [1,2,3] and may also or seldom even exclusively affect the arms. Lipedema shows a prevalence of 5 to 15% [4,5] and is mainly prevalent in female patients [6]. The causes of lipedema are not yet fully understood. It appears that the majority of patients show a positive family history, and, besides this, the involvement of hormonal influences has been discussed [3,7,8]. Diagnostics of lipedema requires a differential understanding of the disease, and lipedema is often misdiagnosed as obesity or lymphedema [9,10]. However, lipedemic tissue is specifically characterized by pain, easy bruising, sensitivity to pressure, and the feeling of tension and tiredness [2,3]. In addition, lipedema tissue does not respond to weight loss interventions, i.e., dietary treatment or exercise [9,11]. Also, more sophisticated diagnostic tools such as dual-energy X-ray absorptiometry or 3D ultrasounds are available [12].

Lipedema can lead to secondary complications such as lymphedema, osteoarthritis, massive impairment in mobility, and aesthetic and psychosocial problems [3,13]. For example, substantial edema inside and outside the legs can cause the knees to bend in or outward and require orthopedic care. These problems can occur in all stages of lipedema [9,11]. Up to now, there is no causal therapeutic concept to cure lipedema [2]. The available treatment options to relieve symptoms and secondary problems such as impairment in mobility and pain can be divided into conservative and surgical strategies. The conservative line of care incorporates combined decongestive therapy (CDT), e.g., compression garments and manual lymphatic drainage [2]. However, the effects are mostly short-term, requiring ongoing treatment within a few days [14]. Surgical treatment includes liposuction to remove the lipedemic tissue [15,16].

### 1.1. Pain and Physical Activity in Lipedema

Pain is known to be the leading symptom of lipedema and has many facets. As with the disease itself, the pathogenesis and etiology of pain in lipedema patients are not yet fully understood [17]. The pain may arise, for instance, from disease-immanent tissue alterations, causing physical impairment [18]. Also, hormonal and genetic aspects are discussed as causes of pain in lipedema. Consistently, pain in lipedema has been associated with estrogen, exaggerated sympathetic signaling, and hyperalgesia [17]. Pain, as the leading symptom of lipedema, can have far-reaching implications. For example, the pain has been associated with physical impairment that can eventually even affect the patients’ basic mobility and the ability to engage in physical activity [3]. With regard to mental health, pain in lipedema patients has been associated with lower levels of quality of life and with elevated levels of depression. Therefore, pain reduction has been defined as a key element in disease treatment [17].

Patients often suffer from obesity as a comorbidity, accentuating the high relevance to enhance weight loss [9,19]. It is well understood that physical activity is a fundamental element of weight regulation. Weight loss in obese patients can considerably reduce the health risks such as diabetes mellitus, hypertension, or cardiovascular diseases [13,20,21]. In addressing weight regulation and mobility in lipedema patients, exercise regimen and physiotherapy have been suggested [22]. Besides numerous health benefits, physical activity is also known to be an important positive influencing factor for mental health [23]. In this context, the involvement of serotonin, β-endorphins, and sympathetic modulation has been associated with the positive effects of physical activity on mood, motivation, and activity levels [24]. Thus, the support of physical activity represents a high-potential option for the effective treatment of depressive disorders. Comparing the effects of physical activity with the administration of antidepressants, engagement in physical activity can lead to similar short-term and even better long-term results in mild to moderate depression [25]. Moreover, the regular practice of physical activity can lead to lower levels of pain sensitivity in women [26]. Despite the high relevance of pain and physical activity in lipedema patients, analyses of specific connections between physical activity and pain in lipedema patients have rarely been targeted yet in previous studies.

### 1.2. Mental Health in Lipedema

Patients suffering from lipedema reported significant impairment in their quality of life (QoL) [3,27]. QoL is an indicator of overall perceived health, subsuming aspects of both physical and mental health [28]. In lipedema patients, both perceived QoL and mental health have been shown to be significantly reduced [13,29,30]. It has been shown that, in particular, pain, swelling, and the sensation of heaviness have been associated with lower levels of QoL in lipedema patients [31]. Also, pain, physical, and aesthetic impairment have been identified as causes of reduced mental health [27].

In addition, not only reduced QoL but also elevated depression levels have been reported in lipedema patients [30,31]. Depression has been identified as one of the top health conditions leading to the loss of life years (DALYs) by the World Health Organization (WHO) and is associated with considerable impairments in autonomy, activity, and the ability to work [32,33]. Reduced activity and motivation levels are known to be key symptoms of depressive disorders [23]. Thus, in turn, reduced motivation can again lead to lower engagement in physical activity. In the context of lipedema patients, a lack of motivation can further impede lipedema patients from engaging in healthy behavior, and, thus, losing weight. Consequently, it is of utmost importance to further elucidate the dynamics and specific interrelations of mental health, depression, and its complex interrelations with physical activity in lipedema patients.

### 1.3. Objectives and Work Program

The current state of evidence indicates that pain, physical activity, and aspects of mental health such as symptoms of depression and QoL stand in a compound interaction. Physical inactivity and the continuous perception of pain could result in a vicious circle-like negative synergy that negatively influences aspects of mental health [13,27,34]. Response surface analysis (RSA) can serve as a method that is based on polynomial regression analysis [35,36]. RSA advances traditional regression analyses, as differentiated effects of the two predictor variables on the outcome variable can be analyzed depending on the degree of their congruence [37]. Thus, RSA is targeting the effects of two variables on an outcome variable considering differentiated effects depending on the predictor variables’ interrelations [36]. In this context, both the interaction effects between the predictor variables (i.e., physical activity and pain) and their (a) main effects on the outcome variable (i.e., mental health) as well as their (b) congruence effects (i.e., the effects on QoL and specifically mental health, given that the predictor variables of pain and physical activity are both high or both low) and the incongruence effects (i.e., the effects on psychological well-being given that pain is high and physical activity is low or pain is low and physical activity is high) including potential synergy effects can be analyzed. Based on the contextual and methodological considerations described above, it is hypothesized:

**Hypothesis 1 (main effect).**  *Higher levels of pain lead to lower levels of mental health*.

**Hypothesis 2 (main effect).**  *Higher levels of physical activity lead to higher levels of mental health*.

**Hypothesis 3 (synergy effect).**  *High levels of pain and low levels of physical activity are synergistically associated with lower levels of mental health*.

## 2. Materials and Methods

### 2.1. Design and Procedure

This study was approved by the Ethics Committee of the Medical Association Westphalia-Lippe and the University of Muenster. Approval Code: 2021-684-f-S. Approval Date: 9 March 2022. Informed consent was obtained from all subjects and/or their legal guardian(s). All methods were carried out in accordance with relevant guidelines and regulations. Participation in this study was voluntary. All patients with suspected lipedema visiting our specialized clinic for an initial consultation were asked to fill in the paper-pencil questionnaires assessing general baseline information. Therefore, age, height, weight, age of symptom onset/diagnosis, family history and stage of lipedema, location and symptoms of pain, previous therapies, comorbidities, QoL, symptoms of depression, and physical activity behavior were assessed. Each patient filled in an individually designed questionnaire prior to their consultation. Only patients later diagnosed with lipedema were included. Patients who had already received a liposuction or any bariatric surgery prior to the consultation were excluded from the study.

### 2.2. Outcome Assessment

Patients were asked to rate their pain in the specific areas (i.e., legs and limbs) that were affected by lipedema. Therefore, a single-item questionnaire was used (“Do you have pain in the affected areas?”). The response was rated on a ten-point Likert scale ranging from one (“none”) to ten (“very strong”). For analysis, the raw score of the single item was used.

Physical activity was assessed via a questionnaire consisting of seven items that were specifically designed for the purpose of this study. Each item represents one dimension (i.e., gym, Nordic walking, swimming, biking, running, others, and none). The patients were asked to define the number of sporting activities per week. For analysis, a sum score of hours per week was calculated.

### 2.3. Mental Health

Mental health (i.e., symptoms of depression and dimensions of QoL) was assessed via the World Health Organization Quality of Life-BREF (WHOQOL-BREF) and the PHQ-9 [28,38] questionnaire. The PHQ-9 is a well-established and validated assessment tool demonstrating high reliability with Cronbach’s α = 0.84 [39]. The PHQ-9 is based on the diagnostics criteria set in the DSM-IV to screen for the presence and severity of depression [38,40]. It entails nine items that are rated on a four-point Likert scale ranging from zero (“none”) to three (“nearly every day”). For analysis, a sum score was calculated according to the guidelines. The sum score is interpreted as follows: Zero to four points represent none to minimal depression; five to nine points represent mild depression; ten to fourteen represent moderate depression; fifteen to nineteen represent moderately severe depression; and twenty to twenty-seven represent severe depression. Thus, higher levels of the PHQ-9 can be interpreted as lower levels of health, respectively.

The WHOQOL-BREF is a proven self-report tool with high validity to assess general QoL. It comprises twenty-six items that are rated on a six-point Likert scale ranging from one (“not at all”) to five (“fully agree”) [28]. The WHOQOL-BREF consists of four subscales including physical health, psychological health, social relationships, and environment. Physical health embodies aspects of mobility, pain, and energy. Psychological health (i.e., mental health) describes negative feelings, self-esteem, and memory/concentration. The social relationships scale represents social support and personal relationships. Environment describes home and physical environment, transport, and financial resources. Each subscale contains six items. Two additional items referring to the total score of QoL are used to assess aspects of general health and overall QoL. The items were recorded, and scores were calculated according to the current guidelines (i.e., mean scores were calculated and multiplied by 4 to facilitate interpretation on a scale ranging from 0 to 100) [28]. The WHOQOL-BREF is a widely used tool to assess the QoL and has demonstrated high validity and reliability with Cronbach’s α ranging from 0.77 to 0.85 [41]. Thus, in total, higher levels of the WHOQOL-BREF questionnaire can be interpreted as higher levels of health, respectively.

### 2.4. Data Analysis

Data analyses were performed via the programming language R [42] with the interface RStudio. To investigate the hypotheses, a complete-case analysis RSA was conducted. In the preliminary inspection of descriptive data, no unplausible data was detected and no data was eliminated. Within the RSA, the full polynomial model is defined as:

$$Z=b_{0}+b_{1}X+b_{2}Y+b_{3}X^{2}+b_{4}XY+b_{5}Y^{2}+\varepsilon$$

in which the centered outcome variable (aspects of mental health and QoL) is regressed on pain in the referring areas (X), physical activity (Y), the squared terms of pain (X^2^) and physical activity (Y^2^), and the cross-product of pain and physical activity (XY). b_0_ to b_5_ represent the unstandardized regression weights. Within the full model described above, more parsimonious (i.e., statistically simpler) models can be identified that fit the data better and that are nested within the full model [36]. The rising ridge model allows the main effects of the predictor variables, forming a tilted ridge (*RR* model), a shifted and tilted ridge (*SRR* model), or a shifted and tilted ridge with an additional rotation (*SRRR* model). The flat ridge model does not allow a main effect, but a mismatch of the predictor variables, again without a tilted but with a shifted ridge (*SSQD* model), or with a shifted and rotated ridge (*SRSQD* model). Furthermore, the additive model, interaction model (*IA* model), and models with single effects of the X-variable (*onlyX* or *onlyX*^2^ models) or Y-variable (*onlyY* or *onlyY*^2^ models) can be identified.

To test and select the best fitting model, model fit indices were compared and evaluated [36]. Therefore, the corrected Akaike Information Criterion (AICc), ΔAICc, and model weight were used to inspect relative fit. The Comparative Fit Index (CFI; with values > 0.95 indicating sufficient model fit), and adjusted *R^2^* were assessed to evaluate absolute model fit. According to Cohen [43], values were interpreted as substantial (*R*^2^*_adj_* = 0.26), moderate (*R*^2^*_adj_* = 0.13), or weak (*R*^2^*_adj_* = 0.02). Practically equivalent models were defined by ΔAICc < 2 and Implausible models were defined by a cumulative weight > 0.95. The models showing the best fit indices were selected for analysis.

Within the three-dimensional visualization of the RSA, the shape of the surface is guided by the lines of congruence and incongruence. Effects of congruence (both predictors are high, or both are low) on the outcome variable are made visible via a line of congruence (LOC). Similarly, the effects of incongruence between the two predictor variables on the outcome variable are visualized via the line of incongruence (LOIC). Regarding the congruence effects described by the LOC, the coefficients a1 and a2 are interpreted. The slope of the LOC is defined as $a_{1}=b_{1}+b_{2}$. A significant coefficient indicates a linear additive effect of the predictor variables. The coefficient $a_{2}=b_{3}+b_{4}+b_{5}$ describes whether there is a curvature on the LOC, i.e., if the effect is linear or curvilinear. Regarding the incongruence effects described by the LOIC, the coefficients a3 and a4 are interpreted. The slope of the LOIC is defined as $a_{3}=b_{1}-b_{2}$. A significant coefficient suggests that the ridge is shifted away from the LOC, i.e., indicating the direction of the predictor variables’ mismatch on the outcome variable. The coefficient $a_{4}=b_{3}-b_{4}+b_{5}$ describes whether there is a curvature on the LOIC, i.e., indicating the degree of the predictor variables’ effect on the outcome variable.

Prior to the main analysis, the stage of lipedema was entered as a moderator variable into linear regression analyses, regressing the outcome variables on pain and physical activity. Therefore, we could examine whether the severity of lipedema (i.e., stage I to III) influenced the relationship between the predictor variables and the outcome variable. As high values of PHQ-9 represent lower levels of health and high levels of WHOQOL-BREF represent higher levels of health, the colors in the RSA visualization were selected as follows. In all analyses, the green color represents higher levels of health, whereas the red color represents lower levels of health.

For RSA analyses, it has been recommended to use a sample size that is two or three times as large as the required sample size for a regression analysis with two predictors [44]. Defining a small to medium effects size of *f*^2^ = 0.05 based on results of previous studies [3], a desired statistical power level of 0.80, and a probability level of α = 0.05, it was estimated that a minimum sample size for a regression analysis with two predictors was *n* = 193 [45]. Thus, a minimum sample size between *n* = 386–579 was required to conduct the RSA.

## 3. Results

All patients enrolled in this study (*n* = 511) were female. Descriptive statistics of the demographic data and results are presented in Table 1. In the preliminary moderator analyses, the stage of lipedema had no effect on the regression of the outcome variables on pain and physical activity in all analyses (*p* > 0.005).

**Figure 1:**
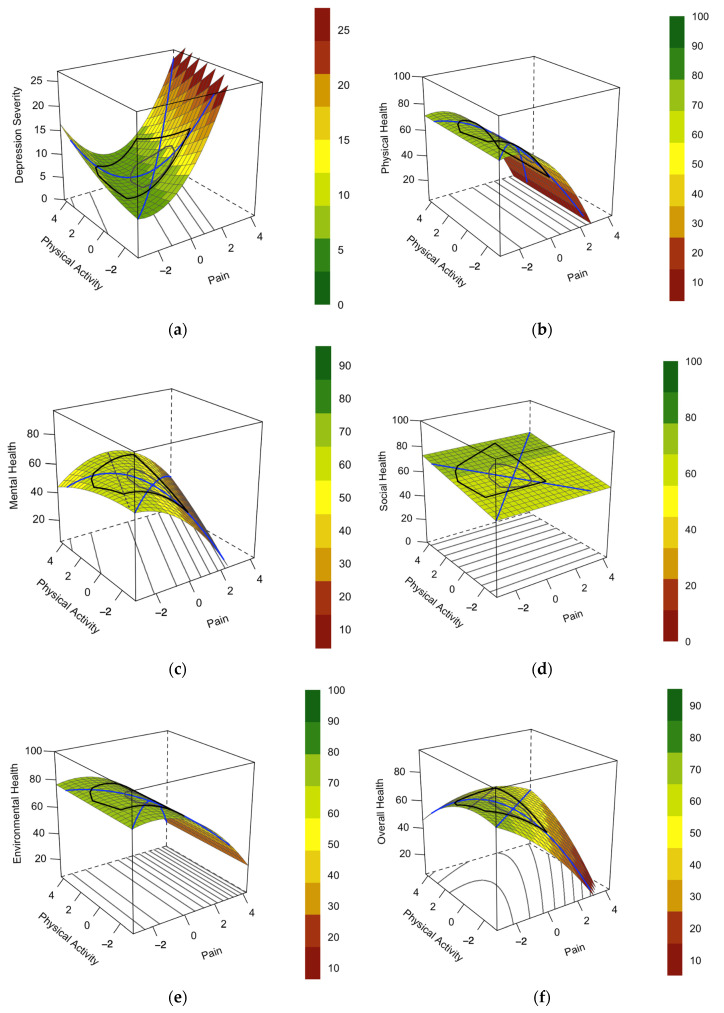
Green color represents higher levels of mental health and QoL; red color represents lower levels of mental health and QoL. (**a**) Depression Severity (*SRSQD* model). (**b**) QoL Physical Health (*onlyX*^2^ model). (**c**) QoL Mental Health (*SRSQD* model). (**d**) QoL Social Health (*onlyY* model). (**e**) QoL Environmental Health (*onlyX*^2^ model). (**f**) QoL Overall Health (*SRR* model).

The model comparisons for all scales are presented in Table 2, and additional plots of the model comparisons are provided in Appendix A. In the main analysis for depression severity measured via the PHQ-9, the *SRSQD* model indicated good model fit and moderate explained variance (see Table 3 and Figure 1). Both physical activity and pain were significant predictors of depression severity. Additionally, a significant linear additive effect on the line of congruence was found. With regard to incongruence effects, a mismatch of the predictor variables (i.e., high levels of pain and low levels of physical activity) positively affects depression severity.

With regard to the physical health scale of the WHOQOL-BREF, the onlyX^2^ model indicated a substantial amount of variance in physical health explained by the predictor variables. A main effect of pain, but not of physical activity on physical health, was found. A significant curvilinear additive effect on the line of congruence was present. A mismatch of the predictor variables (i.e., high levels of pain and low levels of physical activity) negatively affected physical health, hence indicating the presence of incongruence effects.

In the analysis of the mental health scale of the WHOQOL-BREF, the *SRSQD* model indicated weak explained variance and main effects of both pain and physical activity on mental health. No congruence effects were found. In terms of incongruence effects, a mismatch of the predictor variables (i.e., high levels of pain and low levels of physical activity) negatively affected mental health.

Regarding the social health scale of the WHOQOL-BREF, the *onlyY* model indicated no explained variance. No main effect, congruence, or incongruence effect were found. In the analysis of the environmental health scale of the WHOQOL-BREF, the *onlyX*^2^ model indicated weak explained variance and a main effect of only pain but not of physical activity on environmental health. A significant curvilinear additive effect on the line of congruence was present. Looking at the incongruence effects, a mismatch of the predictor variables could be identified but the degree of mismatch on environmental health was not significant.

Concerning the overall health scale of the WHOQOL-BREF, the *SRR* model indicated weak to moderate explained variance and main effects of both pain and physical activity on overall health. A significant linear additive effect on the line of congruence was detectable. In terms of incongruence effects, a mismatch of the predictor variables was found, yet the degree of mismatch on the outcome variable was not significant.

## 4. Discussion

In this study, we hypothesized that higher levels of pain led to lower levels of mental health (Hypothesis 1), that higher levels of physical activity led to higher levels of mental health (Hypothesis 2), and that high levels of pain and low levels of physical activity were synergistically associated with lower levels of mental health (Hypothesis 3). The results of the present study in female lipedema patients provide evidence that pain was the leading entity to all aspects of mental and physical health. Specifically, the remarkable negative effects of pain on physical, mental, environmental, and overall health were found. Also, considerable effects of physical activity on mental health and overall health were found. Thus, Hypotheses 1 and 2 could be confirmed. The results indicate that both pain and physical activity are important influencing factors to explain mental health in lipedema patients. Going beyond these two-dimensional effects, physical activity and pain seem to stand in a synergetic relationship influencing physical, mental, and overall health, confirming Hypothesis 3.

### 4.1. Treatment of Lipedema

No causal treatment option for lipedema has been identified yet. Thus, potential treatment options focus on improving the symptoms that are associated with lipedema [46]. To improve QoL in lipedema patients, effective pain reduction has been understood as a key element [22]. Targeting pain reduction, a multimodal approach including reshaping the affected limbs, compression garments, physical therapy, exercise regimens, diet, and psychological counseling has been suggested.

The support of physical activity in lipedema patients can have beneficial effects on several levels. First, engagement in physical activity can support mobility and weight loss in obesity that often co-occurs in lipedema patients [34]. Second, regular engagement in physical activity has been shown to lead to lower levels of pain sensitivity in healthy women and, thus, could also improve pain sensitivity in women suffering from lipedema [26]. Third, elevated depression levels have been found in lipedema patients [3]. In this context, physical activity can significantly reduce symptoms of depression and therefore could be of high relevance [24].

However, the present findings of this study imply that exercise regimens and physical therapy can be significantly constricted due to massive impairment in mobility and pain. In addition, dietary treatment has been shown to be of limited effectiveness, as lipedemic tissue has hardly been influenced by weight loss intervention [9,11]. Therefore, promising treatment options could focus on pain reduction and reduction in lipedemic tissue.

Following the current State of the Art to reduce lipedemic tissue, most lipedema patients receive conservative therapy [47]. Specifically, the non-invasive CDT incorporates a compression garment, manual lymphatic drainage (MLD), and intermittent pneumatic compression (IPC). However, most of the positive outcomes are of limited value, and some patients even experience an aggravation of symptoms after CDT (i.e., increases in their leg volume) [2,11]. Also, in contrast to obese tissue, lipedema is suspected to be refractory to interventions targeting weight loss via the limitation of calory consumption [9,14]. Consequently, the current literature suggests that liposuction may be a promising invasive approach to attain a long-term reduction in lipidemic tissue [15,16]. Thus, not only primary pain reduction and an increase in the quality of life can be expected.

### 4.2. Mental Health in Lipedema

Targeting reduced QoL and elevated depression levels in lipedema patients, psychological counseling has been suggested [22]. On one hand, treatment of depressive disorders can enhance the reduced motivation and activity levels that are known to be key symptoms of depressive disorders [23]. On the other hand, psychological counseling could also target pain management and the reduction in pain from a subjective perspective. For example, the benefits of interventions such as acceptance and commitment-based therapy (ACT) or mindfulness-based cognitive therapy (MBCT) in the treatment of pain in patients with lipedema have been highlighted [48].

Taken together, pain in the lipidemic tissue can interfere with the opportunity to engage in physical activity. Vice versa, a low pain conception and higher levels of physical activity synergize and lead to higher levels of mental, physical, and overall health. Therefore, pain reduction via reduction in lipidemic tissue appears to be a key aspect in enhancing physical activity, physical health, and mental health in lipedema patients [2,11].

### 4.3. Strengths and Limitations

This study has several methodological strengths. The first is to analyze and contextualize the complex interrelations between physical activity and pain and their effects on aspects of mental health in lipedema patients. Yet, it is based on cross-sectional data and cannot address potential causal relations between the variables. Longitudinal studies are required to further investigate interactions between pain, physical activity, and mental health in lipedema patients.

In this study, physical activity was measured via a tool assessing the number of units per week. This tool provides general information about the amount of physical activity the participants were engaging in per week. Thus, a more detailed assessment of the intensity or differentiation between exercise and physical activity was not possible. The data was obtained from patients presenting in our specialized clinic. The application of such convenience sampling can potentially lead to specific biases in results (e.g., via self-selection).

## 5. Conclusions

Pain was substantially related to lower levels of mental, physical, environmental, and overall health, whereas physical activity positively affected mental, physical, and overall health. Thus, physical activity and pain stand in a complex, vicious circle-like synergetic relationship, influencing physical, mental, and overall health in lipedema patients.

So far, studies focusing on the understanding of treatment and effectiveness, including the complex interrelations between physical and mental health symptoms and health behaviors, are of very high relevance. This study, using sophisticated methodology, had been developed to support filling this gap. Going beyond this study, pain relief and improved mobility resulting from liposuction may provide patients with the novel opportunity to engage in physical activity, and thus to benefit from the overarching and long-term positive effects of physical activity on physical and mental health. Also, the high relevance of supporting psychological consultation is outlined. The results of this study point out that the treatment of lipedema requires a complex and multi-component approach. Responding to this awareness, the inclusion of interventions targeting mental health and pain can have a high impact on the way to best practice treating lipedema patients.

## Figures and Tables

**Table 1:** Descriptive statistics.

Measurement	Measure	Mean	(±SD)	Range
Age	Years	40.16	(±12.45)	16–81
Age at onset of symptoms		19.66	(±10.00)	10–76
Age at diagnosis		36.69	(±11.79)	16–72
BMI	kg/m^2^	33.86	(±7.80)	19.04–64.00
Lipedema	Percentage			
Stage I legs		8.6%	-	-
Stage II legs		57.8%	-	-
Stage III legs		33.6%	-	-
One-Dimensional Tools				
Pain		6.68	(±2.57)	0–10
Physical Activity	hours per week	0.94	(±2.20)	0–10
Depression Severity	PHQ-9	10.84	(±7.26)	0–27
Multi-Dimensional QoL	WHOQOL-BREF			
Physical Health	subscale	54.54	(±22.89)	3.57–100
Mental Health	subscale	51.91	(±21.22)	4.17–95.83
Social Health	subscale	63.69	(±26.14)	0–100
Environmental Health	subscale	71.85	(±17.55)	6.25–100
Overall Health	subscale	60.50	(±18.13)	5.17–95.24

**Table 2:** Model fit indices of the RSA models.

Model	*k*	AIC_c_	∆AIC_c_	Model Weight	Evidence Ratio	CFI	*R* ^2^ *_adj_*	Model *p*
Depression Severity								
**SRSQD**	**5**	**1404.39**	**0.00**	**0.54**	**NA**	**1.00**	**0.18**	<0.001 ***
SRRR	6	1406.42	2.03	0.74	0.00	1.00	0.18	<0.001 ***
SRR	5	1407.47	3.08	0.86	0.00	0.98	0.17	<0.001 ***
QoL^1^ Physical Health								
**onlyX^2^**	**4**	**2089.97**	**0.00**	**0.27**	**NA**	**0.98**	**0.30**	<0.0001 ***
SRSQD	5	2090.19	0.21	0.51	0.00	0.99	0.29	<0.001 ***
SRR	5	2090.51	0.54	0.71	0.00	0.98	0.27	<0.001 ***
QoL^1^ Mental Health								
**SRSQD**	**5**	**2105.60**	**0.00**	**0.30**	**NA**	**1.00**	**0.11**	<0.001 ***
SRR	5	2106.23	0.63	0.51	0.00	1.00	0.10	<0.001 ***
SSQD	4	2106.53	0.93	0.70	0.00	1.00	0.10	<0.001 ***
QoL^1^ Social Health								
null	2	2275.92	0.00	0.15	NA	1.00	0.00	NA
**onlyY**	**3**	**2276.06**	**0.14**	**0.30**	**1.07**	**1.00**	**0.01**	0.04 *
onlyX	3	2276.41	0.49	0.42	1.28	1.00	0.00	0.10
QoL ^1^ Environm. ^2^ Health								
**onlyX^2^**	**4**	**2012.67**	**0.00**	**0.26**	**NA**	**1.00**	**0.08**	<0.001 ***
SRSQD	5	2013.49	0.80	0.44	1.49	1.00	0.08	<0.001 ***
onlyX	3	2013.69	1.00	0.60	1.65	0.97	0.07	<0.001 ***
QoL^1^ Overall Health								
**SRR**	**5**	**1997.31**	**0.00**	**0.29**	**NA**	**1.00**	**0.12**	<0.001 ***
SRSQD	5	1997.77	0.45	0.53	0.00	1.00	0.12	<0.001 ***
SRRR	6	1999.29	1.98	0.64	0.00	1.00	0.12	<0.001 ***

^1^ QoL, Quality of life; ^2^ Environm., Environmental; Bold, models showing the best-fit indices; For the QoL Social Health scale, the null model cannot be calculated and the onlyY model was selected for analysis; * *p* < 0.05; *** *p* < 0.001.

**Table 3:** Coefficients of the RSA models.

	Beta Coefficients					LOC ^3^		LOIC ^4^	
Analysis	*b*_1_	*b*_2_	*b*_3_	*b*_4_	*b*_5_	*a*_1_	*a*_2_	*a*_3_	*a*_4_
Depression	0.46 ***	−0.16 **	0.20 ***	−0.11 *	0.03	1.90 **	0.40	3.91 ***	1.70 ***
QoL ^1^ Physical	−0.61 ***	0.00	−0.17 **	0.00	0.00	−12.40 ***	−2.44 **	−2.44 **	−2.44 **
QoL ^1^ Mental	−0.34 ***	0.15 *	−0.12 *	0.09	−0.03	−3.50	−0.50	−9.14 ***	−3.43
QoL ^1^ Social	0.00	0.09	0.00	0.00	0.00	NA	NA	NA	NA
QoL ^1^ Env. ^2^	−0.33 ***	0.00	−0.11	0.00	0.00	−5.25 ***	−1.30	−5.25 ***	−1.30
QoL ^1^ Overall	−0.35 ***	0.16 *	−0.07	0.11	−0.08	−3.15 *	0.00	−8.09 ***	−3.24

^1^ QoL, Quality of life; ^2^ Env., Environmental; ^3^ LOC, Line of congruence indicating congruence effects; ^4^ LOIC, line of incongruence indicating incongruence effects; * *p* < 0.05; ** *p* < 0.01; *** *p* < 0.001.

**Figure A1:**
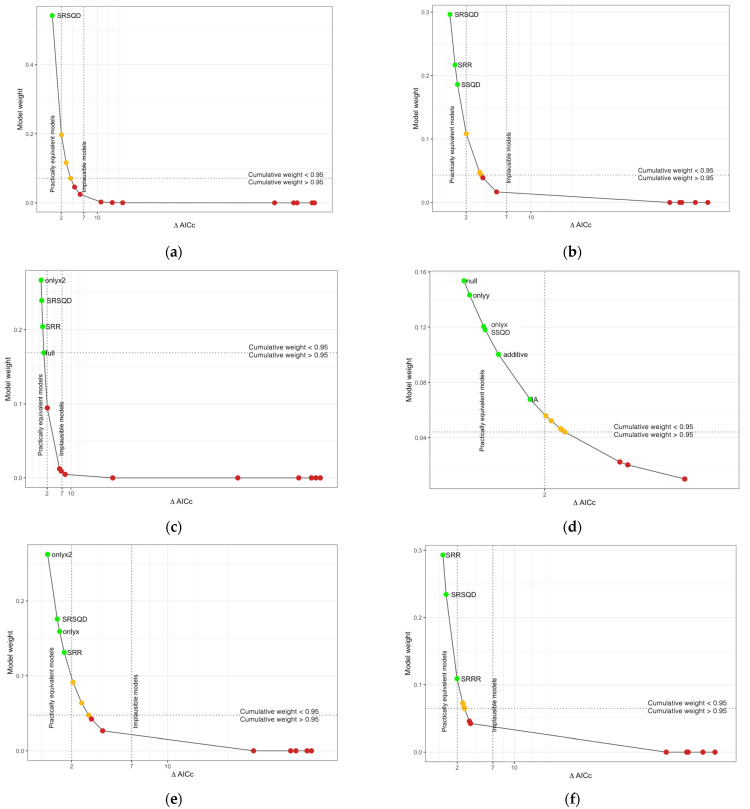
Plots of the model comparisons in the response surface analyses. (**a**) Model comparison for PHQ-9 [38] representing depression severity. (**b**) Model comparison for WHOQOL-BREF [28] mental health subscale. (**c**) Model comparison for WHOQOL-BREF [28] physical health subscale. (**d**) Model comparison for WHOQOL-BREF [28] social health subscale. (**e**) Model comparison for WHOQOL-BREF [28] environmental health subscale. (**f**) Model comparison for WHOQOL-BREF [28] overall health subscale.

## Data Availability

The data was acquired and is stored at the Department of Plastic, Reconstructive and Aesthetic Surgery, Hand Surgery, Fachklinik Hornheide, 48157 Muenster, Germany. A version of the data set that had been anonymized to patient information has been analyzed and can be made available via the corresponding author.
